# SHG/TPEF-based image technology improves liver fibrosis assessment of minimally sized needle biopsies

**DOI:** 10.1007/s12072-019-09955-2

**Published:** 2019-06-11

**Authors:** Bingqiong Wang, Yameng Sun, Jialing Zhou, Xiaoning Wu, Shuyan Chen, Yiwen Shi, Shanshan Wu, Hui Liu, Yayun Ren, Xiaojuan Ou, Jidong Jia, Hong You

**Affiliations:** 1 Liver Research Center, Beijing Key Laboratory of Translational Medicine in Liver Cirrhosis, National Clinical Research Center for Digestive Disease, Beijing Friendship Hospital, Capital Medical University, 95 Yong-an Road, Xi-Cheng District, Beijing, 100050 China; 2 Pathology Department, Beijing Youan Hospital, Capital Medical University, Beijing, China; 3 Hangzhou Choutu Technology, Hangzhou, China

**Keywords:** Liver fibrosis, Liver biopsy, Sampling error, Quantification

## Abstract

**Background and aims:**

Sampling size variability of liver biopsy remains a major limitation in the assessment of liver fibrosis. We aimed to evaluate the diagnostic value of a fully quantitative method (second harmonic generation/two-photon excitation fluorescence, SHG/TPEF based) in “short” liver biopsy samples.

**Methods:**

Liver biopsy samples from chronic hepatitis B (CHB) patients were constructed into “virtual” biopsies with different lengths. The original and “virtual” samples were measured by SHG/TPEF-based technology to obtain qFibrosis score, respectively. Here, ΔqFibrosis was defined as difference of qFibrosis between original biopsy and “virtual” biopsy. Equivalence test was used to compare ΔqFibrosis with the clinically acceptable error (deviation of 0.50) in each group.

**Results:**

In real-world practice, qFibrosis score increased significantly with fibrosis progression in ≥ 1.5-cm-, 1.0–1.5-cm-, and 0.5–1.0-cm-long specimens (*p* < 0.05), compared with ≤ 0.5-cm-long specimens (*p* > 0.05). In virtual biopsy samples with specified length, the equivalence was confirmed in 0.5–1.0-cm- and 1.0–1.5-cm-long specimens (0.27 vs. 0.22, *p* < 0.001), whereas not in ≤ 0.5-cm-long specimens (0.53, *p *> 0.05). The number of cross-linked collagen fibers, the total and aggregated collagen proportionate area, and the collagen strings in number, length, width and perimeter showed excellent consistency with original biopsy samples in 0.5–1.0-cm- and 1.0–1.5-cm-long specimens (ICC > 0.90).

**Conclusions:**

The use of SHG/TPEF-based image technology may give useful suggestive information in evaluation of CHB-related liver fibrosis for the short sample (biopsy length > 0.5 cm).

**Electronic supplementary material:**

The online version of this article (10.1007/s12072-019-09955-2) contains supplementary material, which is available to authorized users.

## Introduction

Liver biopsy has been widely approved as the gold standard in evaluating liver fibrosis for patients with chronic liver disease. However, sampling variability continues to be one of the limitations of liver biopsy in the assessment of liver fibrosis [1–4].

Many studies tried different methods to overcome this limitation. A biopsy specimen with sufficient size has been recommended to minimize sampling error and to improve the diagnostic accuracy of liver biopsies. Whereas, the optimal length remains controversial [5, 6], in an early study, a biopsy length of 1.5 cm was considered adequate [5]. Thereafter, an optimal biopsy sample length of 2.0 cm and 2.5 cm was recommended [4] [7].

However, sufficient sample size cannot be guaranteed for each liver biopsy in clinical practice. Biopsy length data indicate that biopsies smaller than the current recommendations are obtained in over half of the patients [8]. In a landmark clinical study focused on fibrosis reversion in CHB patients who received entecavir therapy, only 60% of biopsies were longer than 1.0 cm [9]. A systematic review reported the mean biopsy length was only 17.7 ± 5.8 mm [10]. Besides, many studies using liver biopsy as gold standard have not shown the data of biopsy length [11, 12].

An effective way to improve the diagnostic value in short liver biopsy samples is needed. In recent years, image morphometric analysis of liver biopsy sample has been applied to quantify the extent of liver fibrosis. qFibrosis (SHG/TPEF based), a structure-based quantitative assessment method, has been recently demonstrated to have a better performance for diagnosis of liver fibrosis compared with traditional collagen proportionate area (CPA) measurement [13]. Due to the comprehensive quantitation of collagen structure features and collagen spatial distribution, qFibrosis was shown to be less sensitive than CPA to sampling size in animal models.

Our aims in this study were: (1) to evaluate the diagnostic value of qFibrosis (SHG/TPEF) measurements for “short” biopsy samples, and (2) to illustrate why this technique is less sensitive to sample size compared to routine CPA measurements.

## Materials and methods

### Liver biopsies

Clinical biopsy samples were retrospectively extracted from a prospective HBV-related fibrosis/cirrhosis cohort study. The study cohort has been described previously [14]. In brief, the inclusions of recruitment were as follows: treatment-naive patients aged 18–65 years, positive for hepatitis B surface antigen (HBsAg) more than 6 months, HBV DNA levels higher than 20,000 IU/mL (positive for HBeAg) or 2000 IU/mL (negative for HBeAg), liver biopsy performed at baseline or week 78 after treatment.

Percutaneous needle biopsies were obtained with real-time ultrasound guidance. Tissue samples were fixed in formalin, embedded in paraffin, sectioned at 5 μm. All samples were stained with hematoxylin and eosin, Masson’s trichrome and reticulin for standard histological assessment. One unstained section from each biopsy was evaluated by SHG/TPEF-based imaging. Biopsy length and fragmentation were documented.

Two senior pathologists (TLW and HL), who were blinded to all data, independently evaluated all the liver biopsy samples using METAVIR scoring system (F0, no fibrosis; F1, portal fibrosis without septa; F2, portal fibrosis with rare septa; F3, numerous septa without cirrhosis; F4, cirrhosis) [15]. Discordant cases will be reviewed again to achieve consensus.

### Image analysis

Biopsies were imaged by second harmonic generation/two-photon excitation fluorescence (SHG/TPEF) microscopy [16]. A total of 101 collagen features were extracted from the SHG images and then the features were normalized by the tissue area. Fifteen collagen architectural features, previously identified as meaningful, were quantified and combined into a single qFibrosis score, as described in our previous study [16].

### Construction of virtual biopsy specimens

**Fig. 1:**
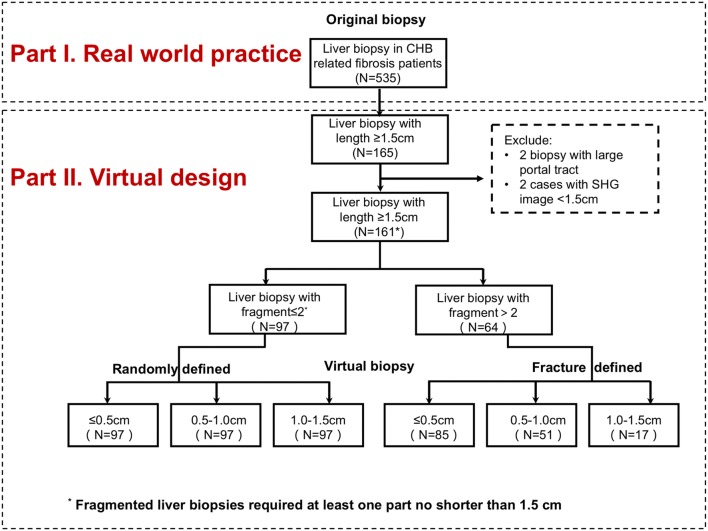
Flow chart of study design. Part I: real-world practice; Part II: construction of virtual liver biopsies

All biopsies ≥ 1.5 cm in length were used to construct virtual biopsy specimens. Samples with ≤ 2 fragments (one of the samples no shorter than 1.5 cm) were used to construct “randomly defined” virtual biopsy specimens. Biopsies with more than two fragments were defined by the fractures (“fracture defined”). Detailed study design is shown in Fig. 1.

For randomly defined virtual biopsy, the first step was to define the starting point, and second step was to decide the specific length from 0.1 to 1.4 cm, all based on a random number generator. Finally, all of virtual biopsies were randomly constructed into different lengths from 0.1 to 1.4 cm long using an image processing tool with precise calibration. Meanwhile, the fragmented biopsies were constructed into virtual biopsies of different lengths from 0.2 to 1.5 cm long. At final, all these samples were classified into four groups: ≤ 0.5-cm, 0.5–1.0-cm, 1.0–1.5-cm, and ≥ 1.5-cm specimens.

The qFibrosis score was determined for each virtual biopsy specimen. The qFibrosis score of the entire tissue section was set as the reference of each case. For each virtual biopsy, the deviation of the qFibrosis score (ΔqFibrosis) was defined as the absolute value of the difference between the score on a virtual biopsy and the score for the entire (reference) biopsy sample. A deviation within 0.50 was set as clinically acceptable error.

### Statistical analysis

Numerical variables were expressed as median with the interquartile range and categorical data as number with frequencies. Intraclass correlation coefficient (ICC) was performed to assess the degree of consistency. ΔqFibrosis was defined as the absolute value of the deviation of qFibrosis scores between a virtual biopsy and the entire biopsy. Equivalence test was used to compare ΔqFibrosis with the clinically acceptable error (deviation of 0.50) in each group. The equivalence between the short biopsy samples and “≥ 1.5-cm”-long specimens was confirmed if ΔqFibrosis fell within 0.5 of the score. Continuous variables were compared using one-way ANOVA or Kruskal–Wallis test. Correlations were evaluated by Spearman’s rank correlation. Equivalence test was performed with SAS 9.4; the other analyses were performed with SPSS 22.0. Two-sided *p* values < 0.05 were considered statistically significant.

## Results

A total of 535 biopsy samples with evaluable fibrosis stage were retrospectively extracted from the prospective cohort study. The prevalence of fibrosis stages was 35.1% for F1 (*n* = 188), 30.1% for F2 (*n* = 161), 19.1% for F3 (*n* = 102) and 15.7% for F4 (*n* = 84).

According to the length of liver biopsy, 30.8% of the samples were ≥ 1.5 cm long (165 cases, median length 1.7 cm), 38.5% were 1.0–1.5 cm long (206 cases, median length 1.2 cm), 26.2% were 0.5–1.0 cm long (140 cases, median length 0.9 cm), and 4.5% were ≤ 0.5 cm long (24 cases, median length 0.5 cm). A total of 165 (30.8%) samples with biopsy length ≥ 1.5 cm were considered as qualified biopsy samples. The remaining biopsies (69.2%) < 1.5 cm were defined as unqualified or “short” biopsy samples. The distribution of fibrosis stage according to the biopsy length is shown in Table 1.

**Table 1:** The distribution of biopsy length in biopsy specimens quantified by SHG/TPEF technology-based microscope and evaluated by Metavir stage

Biopsy length group	Total	Proportion of METAVIR fibrosis stage, *n* (%)			
		*F*1	*F*2	*F*3	*F*4
≤ 0.5 cm, *n* (%)	24 (4.5)	8 (1.5)	6 (1.1)	7 (1.3)	3 (0.6)
0.5–1.0 cm, *n* (%)	140 (26.2)	62 (11.6)	35 (6.5)	23 (4.3)	20 (3.7)
1.0–1.5 cm, *n* (%)	206 (38.5)	58 (10.8)	67 (12.5)	38 (7.1)	43 (8.0)
≥ 1.5 cm, *n* (%)	165 (30.8)	60 (11.2)	53 (9.9)	34 (6.4)	18 (3.4)
Total, *n* (%)	535 (100)	188 (35.1)	161 (30.1)	102 (19.1)	84 (15.7)

Data presented as number (percent)

### Influence of biopsy length on the evaluation for fibrosis by qFibrosis for “short” samples in clinical practice: qFibrosis was good, especially in biopsy samples longer than 0.5 cm

**Fig. 2:**
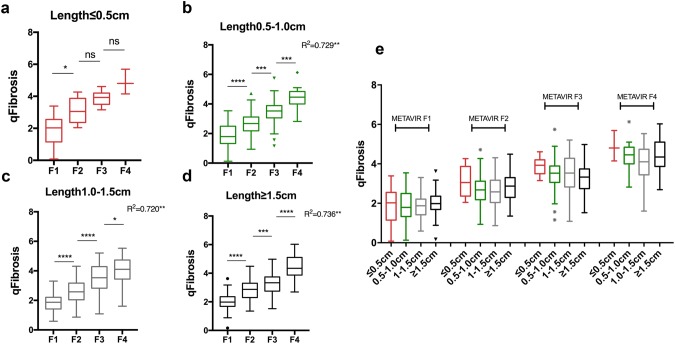
Correlations of qFibrosis scores and METAVIR fibrosis score in original biopsies based on length. **a** Correlations of qFibrosis with METAVIR fibrosis stage in ≤ 0.5-cm-long specimens (*p* > 0.05). **b** Correlations of qFibrosis with METAVIR fibrosis stage in 0.5–1.0-cm-long specimens (*p* < 0.001, γs = 0.729). **c** Correlations of qFibrosis with METAVIR fibrosis stage in 1.0–1.5-cm-long specimens (*p* < 0.001, γs = 0.720). **d** Correlations of qFibrosis with METAVIR fibrosis stage in ≥ 1.5-cm-long specimens (*p* < 0.001, γs = 0.736). **e** Distribution of qFibrosis with fibrosis progression according to biopsy length. ****p < 0.0001

First, we investigated the influence of biopsy length on the value of qFibrosis in differentiating fibrosis staging in clinical practice (Fig. 1, Part I). With fibrosis progression, qFibrosis score increased significantly both in ≥ 1.5-cm, 1.0–1.5-cm, and 0.5–1.0-cm specimens (*p *< 0.05), compared with ≤ 0.5-cm specimens (*p *>0.05) (Fig. 2a–d). As shown in Fig. 2a–d, the Spearman’s correlation coefficient was 0.729, 0.720, 0.736 in 0.5–1.0-cm, 1.0–1.5-cm, and ≥ 1.5-cm specimens, respectively, all *p* values < 0.05. qFibrosis had a significant correlation with fibrosis stage in biopsy samples longer than 0.5 cm.

**Fig. 3:**
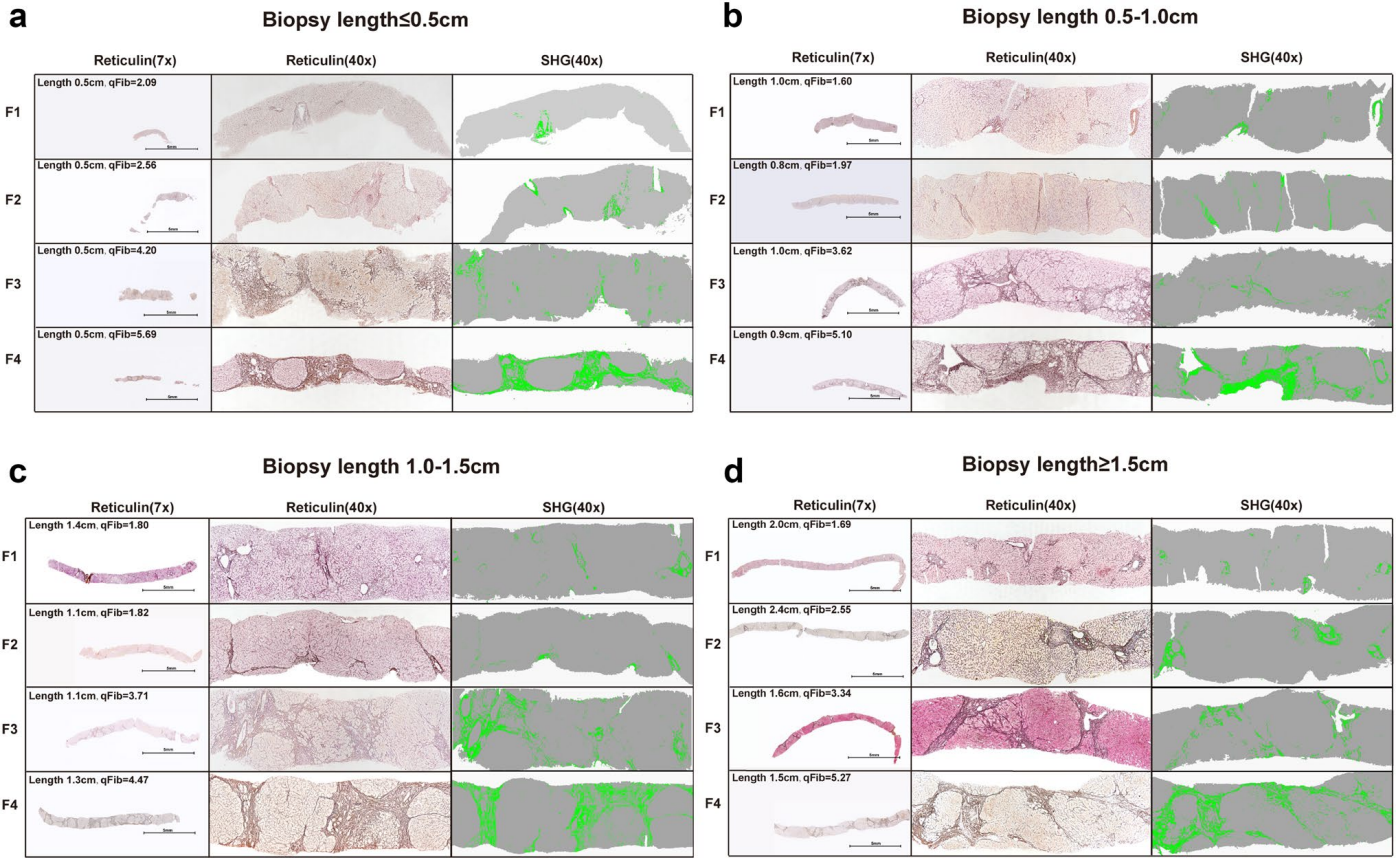
Representative liver biopsy samples with various severity of fibrosis according to different biopsy length. The right panel showed the preview of liver biopsy (Reticulin, 7X). The middle panel indicated the typical histological feature (reticulin, 40X). The left panel showed the image analysis results of liver biopsy by SHG/TPEF-based technology (pseudo-color, green stands for collagen fiber acquired by SHG signal, gray stands for parenchyma acquired by TPEF signal). **a** The reticulin staining and SHG/TPEF image in ≤ 0.5-cm-long specimens. qFibrosis score of F1–F4 stage was 2.09, 2.56, 4.20 and 5.69, respectively. **b** The reticulin staining and SHG/TPEF image in 0.5–1.0-cm-long specimens. qFibrosis score of F1–F4 stage was 1.60, 1.97, 3.62 and 5.10, respectively. **c** The reticulin staining and SHG/TPEF image in 1.0–1.5-cm-long specimens. qFibrosis score of F1–F4 stage was 1.80, 1.82, 3.71 and 4.47, respectively. **d** The reticulin staining and SHG/TPEF image in ≥ 1.5-cm-long specimens. qFibrosis score of F1–F4 stage was 1.69, 2.55, 3.34 and 5.27, respectively

Interestingly, Fig. 2e shows the qFibrosis score might be higher in ≤ 0.5-cm-long specimens than the other three groups in each fibrosis stage, though there was no significant difference. Figure 3 illustrates the images of representative biopsy samples with different sample size in each fibrosis stage. The qFibrosis score was slightly higher in ≤ 0.5-cm specimens (Fig. 3a) than the other three groups at the same fibrosis stage (Fig. 3b–d).

### The influence of biopsy length on qFibrosis score by comparing “virtual” biopsy samples with original biopsy

Then, to clarify the diagnostic value of qFibrosis score in differentiate fibrosis influenced by biopsy length, a total of 444 virtual biopsy samples were obtained from 161 qualified biopsies (≥ 1.5-cm-long specimens, excluding two biopsies with large portal tract, two cases due to pathological process made the effective length of biopsy in SHG image < 1.5 cm). The process of virtual liver biopsy construction is shown in Fig. 1, Part II.

qFibrosis score was less sensitive to biopsy length than CPA. For “randomly defined” virtual biopsy specimens, the relative deviation of qFibrosis against the original sample was gradually decreased from ≤ 0.5-cm-long specimens to 1.0–1.5-cm-long specimens and was smaller than that of CPA for each group (all *p* values < 0.001, Supplementary Fig. 1).

**Fig. 4:**
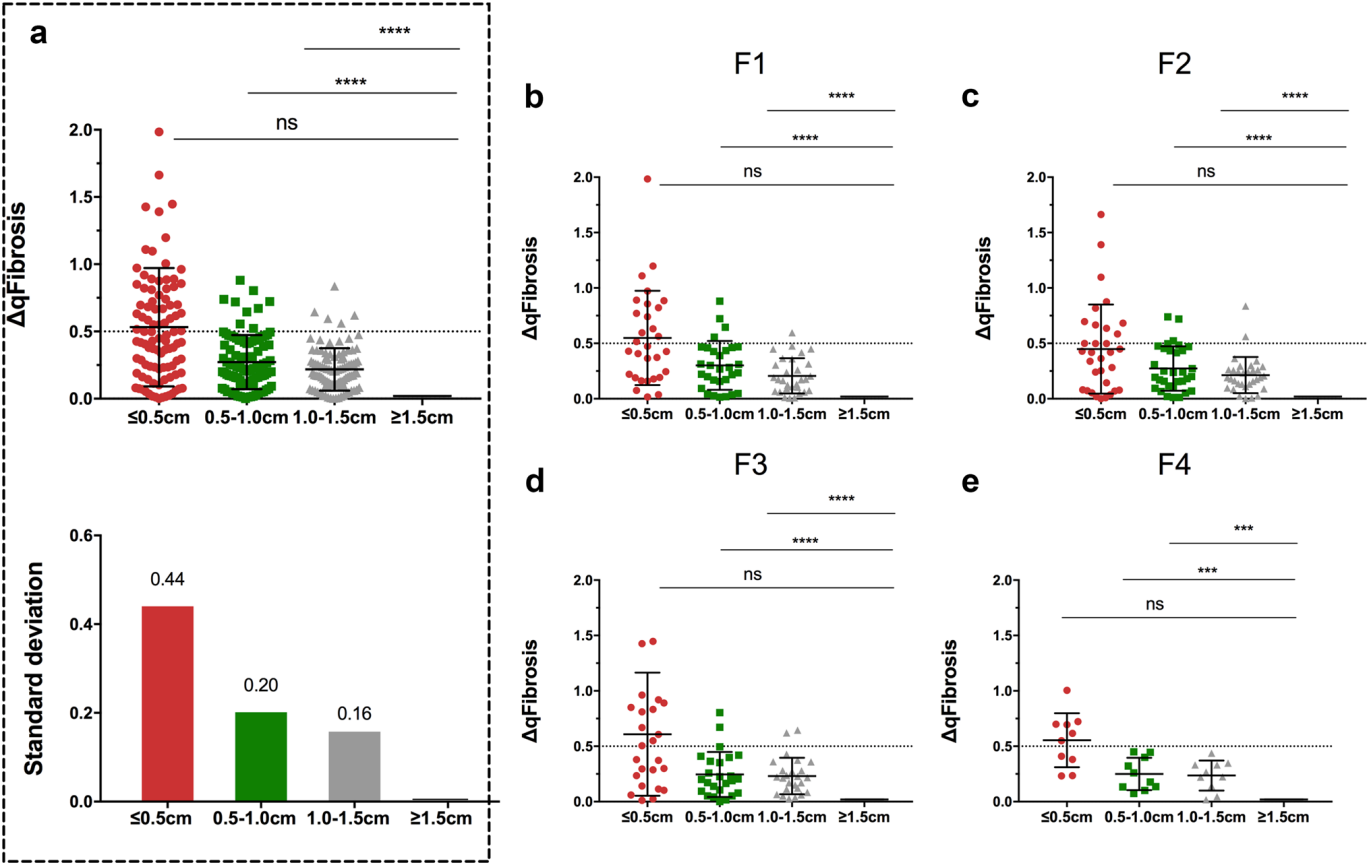
Box plot showing the deviation (absolute value of ΔqFibrosis) in randomly defined virtual biopsies. ΔqFibrosis in ≥ 1.5-cm group, shown as 0, means a reference value for the other three groups. **a** The distribution of deviation in virtual biopsies with all spectrum of fibrosis stage according to biopsy length. ****p < 0.0001. **b** The distribution of deviation in virtual biopsies with METAVIR stage F1 according to biopsy length. ****p < 0.0001. **c** The distribution of deviation in virtual biopsies with METAVIR stage F2 according to biopsy length. ****p < 0.0001. **d** The distribution of deviation in virtual biopsies with METAVIR stage F3 according to biopsy length. ****p < 0.0001. **e** The distribution of deviation in virtual biopsies with METAVIR stage F4 according to biopsy length. ***p < 0.001. ****p < 0.0001; NS, *p* > 0.05

In “randomly defined” virtual biopsy samples, qFibrosis score for the assessment of liver fibrosis was equivalent between 0.5-, 1.0-cm and ≥ 1.5-cm specimens, as well as 1.0–1.5-cm and ≥ 1.5-cm specimens (all *p* values < 0.001), whereas not in ≤ 0.5-cm specimens. The proportion of absolute value of ΔqFibrosis within 0.50 was 55.7, 90.7 and 94.8% in ≤ 0.5-cm, 0.5–1.0-cm and 1.0–1.5-cm specimens, respectively. The distribution of absolute deviation is shown in Fig. 4. In total, the mean absolute deviation was 0.53 in ≤ 0.5-cm specimens, 0.27 in 0.5–1.0-cm specimens, and 0.22 in 1.0–1.5-cm specimens. The standard deviation decreased as biopsy size increased (SD = 0.44, 0.20, 0.16 in ≤ 0.5-cm, 0.5–1.0-cm and 1.0–1.5-cm specimens, respectively). According to the subgroup analysis, the difference existed from F1 through F4 stages (Fig. 4b–e).

qFibrosis could distinguish the METAVIR stages well both in 0.5–1.0-cm and 1.0–1.5-cm specimens (AUC: 0.78–0.88), as shown by ROC curve analysis (Supplementary Fig. 2).

**Fig. 5:**
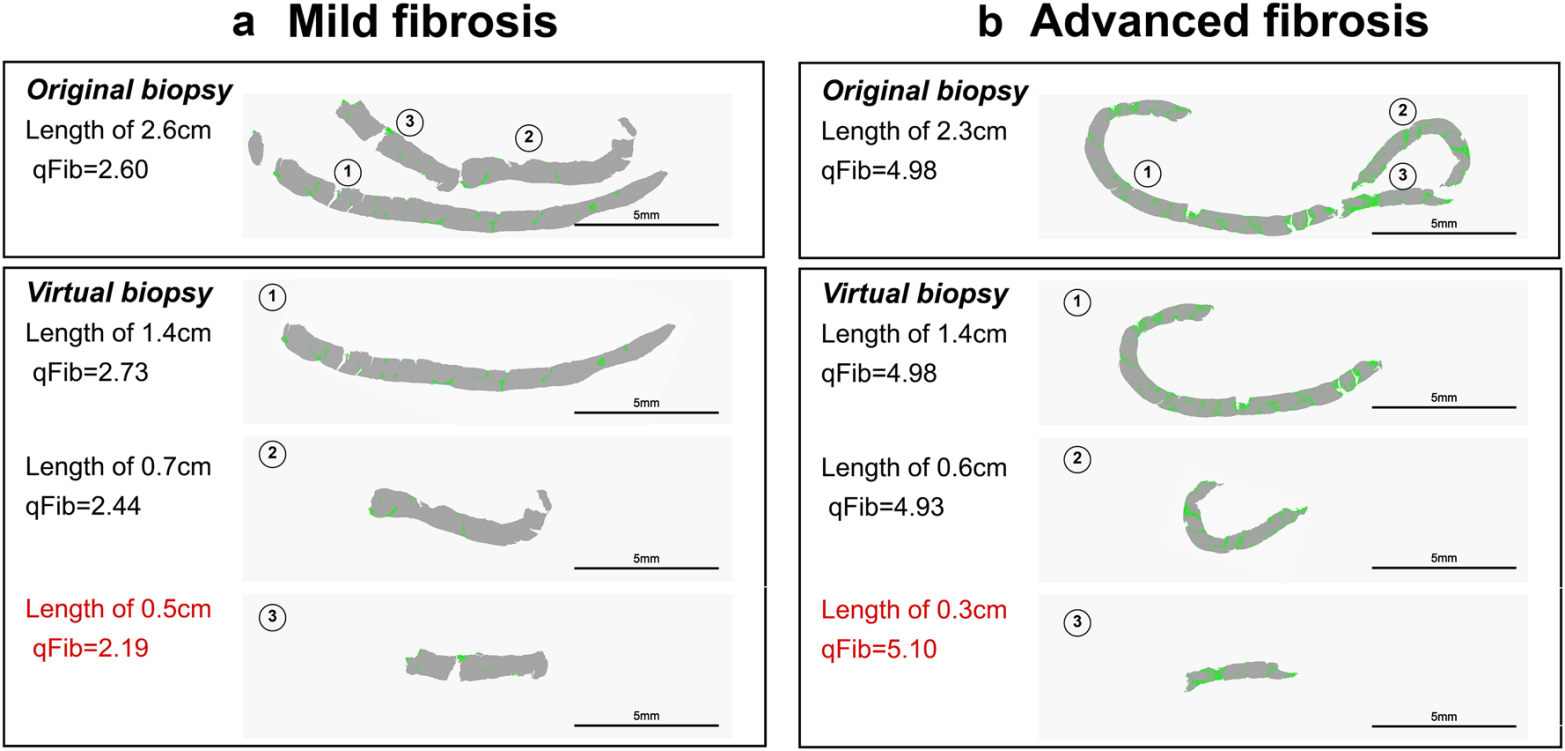
Processed imaging of liver biopsies longer than 1.5 cm with fragmentation showing the deviation in ≤ 0.5-cm-long specimen was higher than the other two groups. Virtual biopsies were constructed along the fractures (pseudo-color, green stands for collagen fiber acquired by SHG signal, gray stands for parenchyma acquired by TPEF signal). **a** Representative biopsy samples with mild fibrosis (F1). qFibrosis score of the original biopsy is 2.60. After virtual image selection, in biopsy longer than 0.5 cm, the qFibrosis score is maintained at 2.73 and 2.44, respectively. In the smallest fragmented biopsy samples shorter than 0.5 cm, the qFibrosis score decreased to 2.19. Scale bar = 5 mm. **b** Representative biopsy samples with advanced fibrosis (F3). qFibrosis score of the original biopsy is 4.98. After image selection, in biopsy longer than 0.5 cm, the qFibrosis score is maintained at 4.98 and 4.93, respectively. In the smallest fragmented biopsy samples shorter than 0.5 cm, the qFibrosis score increased to 5.10. Scale bar = 5 mm

For comparison, the distribution of absolute value of ΔqFibrosis in virtual samples acquired from fractured liver biopsies is shown in Supplementary Fig. 3. qFibrosis score was equivalent between 0.5-, 1.0-cm and ≥ 1.5-cm specimens, as well as 1.0–1.5-cm and ≥ 1.5-cm specimens, whereas not in ≤ 0.5-cm specimens. Examples of representative liver samples are shown in Fig. 5. As for mild fibrosis, the qFibrosis score was 2.60, and it remains at 2.44–2.73 in the longer fragments part of these samples, and reduced to 2.19 in samples shorter than 0.5 cm. In advanced fibrosis, we can also see the same trend. The absolute value of ΔqFibrosis was higher when sample shorter than 0.5 cm.

Therefore, qFibrosis is relatively accurate in the evaluation of liver fibrosis in short samples if the length is greater than 0.5 cm.

### The reason that qFibrosis overcomes the drawbacks of small sample size: additional parameters are considered compared to routine collagen proportionate area calculation

**Fig. 6:**
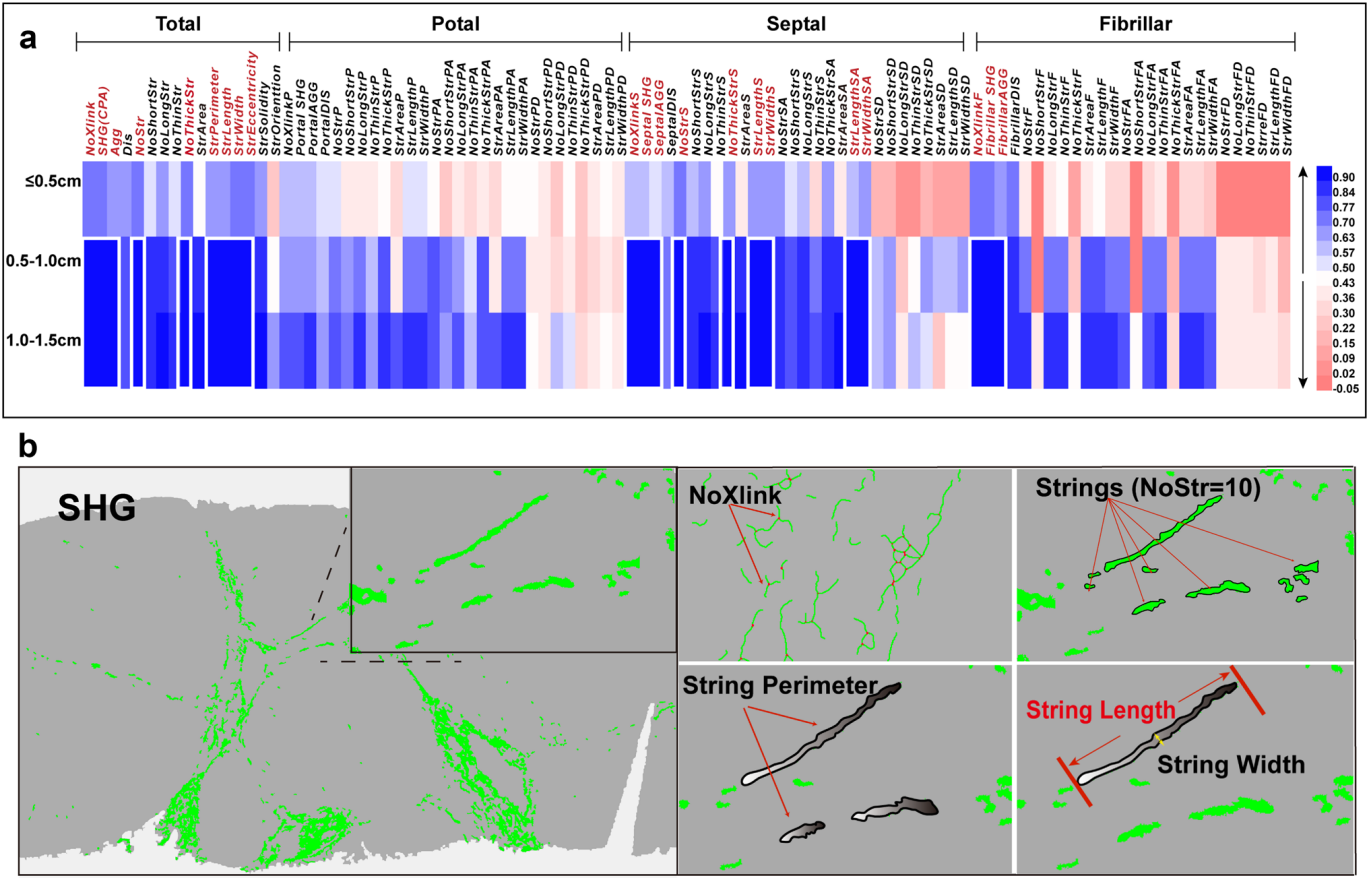
Heatmap and quantitative diagram of morphological parameters by SHG/TPEF technology in virtual biopsies. **a** Heatmap of the intraclass correlation efficient between “unqualified” biopsy samples and “qualified” biopsy samples of each quantitative parameters; **b** Quantitative diagrams of many of the morphological features which were less susceptible to biopsy length, including collagen proportion area by, length, width and contour perimeter of collagen string, cross-linked collagen strings. SHG, total collagen proportionate area quantified by SHG/TPEF; Agg, aggregated collagen; Dis, distributed collagen; NoXlink, number of cross-linked collagen strings; NoStr, number of collagen strings; StrLength, the length of collagen strings; StrWidth, the width of collagen strings; StrEccentricity, the eccentricity of collagen strings; StrSolidity, the solidity of collagen strings; StrPerimeter, the perimeter of collagen strings

To further explore why qFibrosis scores show good performance in evaluating fibrosis, we investigated the value of the additional parameters included in the calculation of the qFibrosis scores. Intraclass correlation coefficient of a possible 98 candidate parameters are shown in a heatmap (Fig. 6a). Among these candidate parameters, in samples of 0.5–1.0 cm and 1.0–1.5 cm length, 21 of these parameters had good consistency with results from larger specimens (≥ 1.5 cm) (ICC > 0.90, Supplementary Table 1). Quantitative diagrams of many of the parameters are shown in Fig. 6b. In summary, the most consistent parameters were: the number of cross-linked collagen fibers, the total and aggregated collagen proportionate area, the number, length, width, and perimeter of collagen strings. It should be noted that the definition of cross-linked collagen fibers in the qFibrosis nomenclature bears no relation to collagen cross-linking at the molecular level used elsewhere in the hepatic fibrosis literature; it refers to the physical contacts of the fibers.

Considering the spatial distribution of the respective collagen patterns, portal area is susceptible to the change of biopsy length, followed by fibrillar area and septal area. AUROC of the consistent parameters in septal and fibrillar areas are shown in Supplementary Table 2. Compared with portal area, quantitative characters in the septal and fibrillar areas could be meaningful in diagnosing the severity of liver fibrosis. Detailed change of CPA in each area, the representative feature, is shown in Supplementary Fig. 4. The change percentage of CPA of total area, septal area and fibrillar area has no significant difference in 0.5–1.0-cm and 1.0–1.5-cm groups, whereas, not in portal area.

Among the 21 most consistent parameters, 9 are used to establish qfibrosis score, accounting for 60% of parameters of qFibrosis scores, perhaps illustrating why qFibrosis can minimize errors induced by small sample size.

## Discussion

In this study, we demonstrated that biopsy samples longer than 0.5 cm are able to deliver a reliable quantitative assessment of fibrosis using the fully quantitative method of qFibrosis. The histological features in septal and fibrillar area were insusceptible to sample size, illustrating the reason why qFibrosis has an advantage in resolving the issue of sampling error.

The impact of biopsy length on quantitative fibrosis score (qFibrosis) in real-world practice of CHB patients was first analyzed. qFibrosis showed a very good performance in reflecting the severity of fibrosis in “short” biopsy samples if it is longer than 0.5 cm. It is not clear whether this good performance is caused by underestimation of METAVIR fibrosis stage or improved detection of fibrosis with the qFibrosis technology. To clarify this issue, we constructed “virtual biopsy samples” from actual biopsies ≥ 1.5 cm in length. These “virtual” biopsies simulated the so-called “short” biopsy specimens. The deviation and consistency of each biopsy were individually calculated by comparing with the biopsies longer than 1.5 cm. According to our analysis, the absolute value of ΔqFibrosis maintained a low level in liver biopsies if they were at least 0.5 cm in length. Similar to our results, another study has shown recently that qFibrosis was less sensitive to sample size than CPA [13].

The reason why this SHG/TPEF-based image analysis could reliably reflect the severity of liver fibrosis in small biopsies is the consistency of the established quantitative parameters between long and short biopsy samples. In our study, we found the stable features included: the number of cross-linked collagen fibers, the total and aggregated collagen proportionate area, the length, width, area and perimeter of collagen strings in total. qFibrosis, SHG/TPEF-based image technology, incorporates the multiple spatial architectural collagen features, giving the reason why qFibrosis can minimize errors induced by small sample size.

Interestingly, when considering the spatial characteristics of collagen pattern, features in septal and fibrillar areas were less sensitive to a reduced biopsy length, compared to portal features. Quantitative characters in the septal and fibrillar areas are meaningful in diagnosing the severity of liver fibrosis compared with the features in portal area. This sensitivity in small biopsies may be explained by reduced portal tract number while histological features in septal and fibrillar areas remain relatively constant because of their distributed location.

These favorable results with qFibrosis are in contrast to those from studies with traditional methods demonstrating substantial sampling error [4, 6, 7, 17, 18]. For these methods, a relatively generous 2-cm-long sample is now recommended [8, 19], leading to increased peri-operative risk [10, 20]. Traditional methods are also affected by lack of expertise and poor interobserver agreement, while qFibrosis is totally independent of these effects [21].

Traditional staging methods have been compared to standard digital image analysis, focusing on the issue of sampling error. [22] In a study of cirrhotic transplant tissue, Hall et al. concluded that, to achieve the 75% probability that CPA of a virtual biopsy will be within 5% of the reference CPA, a length of 15–20 mm was required [22]. This requirement does not show an advantage of CPA over traditional histological analysis in the issue of sampling error. Our results suggest that qFibrosis methodology is more resistant to sampling error compared to CPA measurements by standard digital image analysis methods and the benefits are extended across the fibrosis range of F1–F4.

The clinical implications of these results might be profound, as shown in Supplementary Fig. 5. In the past, the guidelines have recommended liver biopsy length be at least 1.5 cm, to avoid sampling error, while our data with qFibrosis suggest that 0.5 cm may be sufficient for this purpose.

The strengths of our study are as follows: (1) the quantitative method we used is more sensitive and specific than traditional digital image analysis. It has been demonstrated to have better diagnostic value for discriminating adjacent stages even in early fibrosis stage compared with traditional digital image analysis. (2) The samples used in our study were liver biopsies, as opposed to samples obtained from liver transplantation with a limited range of stages. The simulation of “short” biopsy sample was close to the actual possibility. (3) Our cohort, including 165 biopsies, was larger than the other studies focused on the same issue.

There are still some limitations of our study. First, liver biopsy sample of cirrhosis is more easily to be fragmented, which is a major obstacle in the diagnosis of cirrhosis. However, the proportion of cirrhotic specimens is relatively small in our study, so the diagnostic value of qFibrosis in short cirrhotic samples needs to be further verified in larger number of samples. Second, all the liver biopsies were obtained from CHB patients in our study. The histological pattern of fibrosis from other etiologies may be different from CHB patients, indicating the application of qFibrosis in “short” biopsies other than CHB needs to be further explored.

Despite these limitations, our study still demonstrated that SHG/TPEF-based technology, coupled with additional parameters discovered by machine learning (qFibrosis scores) showed good performance in the evaluation of CHB-related liver fibrosis in short samples down to a lower limit of 0.5 cm in length. Future studies would be of great interest to search for further improvements in the diagnostic value of qFibrosis using small “unqualified” liver biopsies in the assessment of liver fibrosis.

## Electronic supplementary material

Below is the link to the electronic supplementary material.

Supplementary material 1 (DOCX 18 kb)

Supplementary material 2 (DOCX 16 kb)

Supplementary material 3 (TIFF 745 kb)

Supplementary material 4 (TIFF 1734 kb)

Supplementary material 5 (TIFF 1635 kb)

Supplementary material 6 (TIFF 2289 kb)

Supplementary material 7 (TIFF 3384 kb)
