# Supplementary material for: SHG/TPEF-based image technology improves liver fibrosis assessment of minimally sized needle biopsies
Source: Hepatol Int. 2019 Jun 11;13(4):501–9. doi: 10.1007/s12072-019-09955-2 (PMC6661026; doi:10.1007/s12072-019-09955-2)
Supplement: Supplementary file 1 — Supplementary material 1 (DOCX 18 kb) [file 12072_2019_9955_MOESM1_ESM.docx]

**Supplementary Table 1.** **Intraclass correlation coefficient of the consistent quantitative features in septal area and fibrillar area.**

| **Parameters** | **≤ 0.5cm** | **0.5-1.0 cm** | **1.0-1.5 cm** |
| --- | --- | --- | --- |
| **Total area** |  |  |  |
| **NoXlink** | 0.732 | 0.967 | 0.973 |
| **CPA-SHG** | 0.710 | 0.970 | 0.970 |
| **StrPerimeter** | 0.643 | 0.935 | 0.968 |
| **Agg** | 0.670 | 0.960 | 0.960 |
| **StrLength** | 0.663 | 0.932 | 0.952 |
| **StrWidth** | 0.711 | 0.932 | 0.947 |
| **StrEccentricity** | 0.704 | 0.906 | 0.931 |
| **NoThickStr** | 0.710 | 0.910 | 0.930 |
| **NoSt** | 0.710 | 0.910 | 0.930 |
| **Septal area** |  |  |  |
| **NoXlinkS** | 0.622 | 0.951 | 0.956 |
| **Septal CPA-SHG** | 0.567 | 0.956 | 0.954 |
| **SeptalAGG** | 0.535 | 0.954 | 0.947 |
| **StrWidthS** | 0.673 | 0.916 | 0.943 |
| **StrLengthS** | 0.660 | 0.923 | 0.942 |
| **StrWidthSA** | 0.642 | 0.909 | 0.929 |
| **StrLengthSA** | 0.619 | 0.916 | 0.922 |
| **NoStrS** | 0.711 | 0.919 | 0.913 |
| **NoThickStrS** | 0.686 | 0.914 | 0.905 |
| **Fibrillar area** |  |  |  |
| **Fibrillar CPA-SHG** | 0.732 | 0.952 | 0.953 |
| **FibrillarAGG** | 0.659 | 0.930 | 0.921 |
| **NoXlinkF** | 0.711 | 0.946 | 0.954 |

**Abbreviation:** SHG/ Agg, total/aggregated collagen proportionate area quantified by SHG/TPEF; StrPerimeter: perimeter of collagen strings; NoStr/ NoStrS/ NoStrF, number of collagen strings in total/septal/fibrillar area; StrLength/ StrLengthS/ StrLengthSA, the length of total/ aggregated collagen strings in total/ septal area; StrLength/ StrLengthS the length of collagen strings in total/septal area; StrWidth/ StrWidthS/ StrWidthSA, the width of total/aggregated collagen strings in total/septal area; NoThickStr/ NoThickStrS, number of thick strings in total/septal area; NoXlink/ NoXlinkS/ NoXlinkF, number of cross-linked collagen strings in total/septal/fibrillar area.
