# Supplementary material for: SHG/TPEF-based image technology improves liver fibrosis assessment of minimally sized needle biopsies
Source: Hepatol Int. 2019 Jun 11;13(4):501–9. doi: 10.1007/s12072-019-09955-2 (PMC6661026; doi:10.1007/s12072-019-09955-2)
Supplement: Supplementary file 2 — Supplementary material 2 (DOCX 16 kb) [file 12072_2019_9955_MOESM2_ESM.docx]

**Supplementary Table 2. Diagnostic value of the consistent quantitative features in septal area and fibrillar area.**

|  | **Significant fibrosis** | | | **Advanced fibrosis** | | | **Cirrhosis** | | |
| --- | --- | --- | --- | --- | --- | --- | --- | --- | --- |
|  | **≤ 0.5cm** | **0.5-1.0 cm** | **1.0-1.5 cm** | **≤ 0.5cm** | **0.5-1.0 cm** | **1.0-1.5 cm** | **≤ 0.5cm** | **0.5-1.0 cm** | **1.0-1.5 cm** |
| **Portal CPA-SHG*** | 0.52  (0.40, 0.64) | 0.50  (0.38, 0.62) | 0.56  (0.45, 0.68) | 0.50  (0.38, 0.63) | 0.45  (0.32, 0.59) | 0.53  (0.40, 0.66) | 0.68  (0.46, 0.90) | 0.62  (0.40, 0.84) | 0.62  (0.39, 0.84) |
| **Septal CPA-SHG** | 0.73  (0.63, 0.83) | 0.84  (0.76, 0.92) | 0.82  (0.73, 0.91) | 0.68  (0.56, 0.79) | 0.76  (0.66, 0.86) | 0.76  (0.66, 0.86) | 0.85  (0.71, 0.99) | 0.88  (0.76, 1.00) | 0.89  (0.78, 1.00) |
| **SeptalAGG** | 0.74  (0.64, 0.85) | 0.83  (0.74, 0.91) | 0.81  (0.72, 0.90) | 0.68  (0.57, 0.79) | 0.76  (0.65, 0.86) | 0.75  (0.65, 0.86) | 0.85  (0.72, 0.98) | 0.89  (0.79, 1.00) | 0.90  (0.79, 1.00) |
| **NoStrS** | 0.76  (0.66, 0.86) | 0.84  (0.76, 0.92) | 0.86  (0.78, 0.94) | 0.77  (0.67, 0.87) | 0.84  (0.75, 0.93) | 0.85  (0.77, 0.93) | 0.84  (0.72, 0.96) | 0.92  (0.84, 1.00) | 0.92  (0.85, 1.00) |
| **NoThickStrS** | 0.77  (0.67, 0.87) | 0.82  (0.73, 0.90) | 0.84  (0.75, 0.92) | 0.73  (0.62, 0.84) | 0.79  (0.69, 0.89) | 0.81  (0.72, 0.90) | 0.84  (0.72, 0.95) | 0.86  (0.74, 0.99) | 0.88  (0.77, 0.99) |
| **StrLengthS** | 0.71  (0.60, 0.82） | 0.80  (0.72, 0.89) | 0.84  (0.76, 0.92) | 0.71  (0.60, 0.82) | 0.80  (0.70, 0.90) | 0.81  (0.72, 0.90) | 0.85  (0.73, 0.97) | 0.94  (0.88, 1.00) | 0.94  (0.87, 1.00) |
| **StrWidthS** | 0.73  (0.63, 0.83) | 0.81  (0.72, 0.90) | 0.84  (0.75, 0.92) | 0.71  (0.60, 0.82) | 0.79  (0.69, 0.89) | 0.80  (0.71, 0.90) | 0.85  (0.75, 0.96) | 0.90  (0.81, 0.99) | 0.91  (0.82, 1.00) |
| **StrLengthSA** | 0.71  (0.60, 0.82) | 0.78  (0.68, 0.87) | 0.85  (0.77, 0.94) | 0.70  (0.59, 0.81) | 0.80  (0.69, 0.90) | 0.80  (0.71, 0.90) | 0.82  (0.70, 0.94) | 0.94  (0.88, 1.00) | 0.93  (0.87, 1.00) |
| **StrWidthSA** | 0.74  (0.64, 0.84) | 0.79  (0.70, 0.88) | 0.86  (0.78, 0.94) | 0.70  (0.59, 0.82) | 0.79  (0.68, 0.89) | 0.81  (0.71, 0.90) | 0.84  (0.73, 0.95) | 0.91  (0.83, 0.99) | 0.92  (0.84, 1.00) |
| **NoXlinkS** | 0.76  (0.66, 0.86) | 0.85  (0.77, 0.93) | 0.85  (0.77, 0.93) | 0.71  (0.59, 0.82) | 0.79  (0.69, 0.88) | 0.80  (0.70, 0.89) | 0.84  (0.69, 0.98) | 0.85  (0.71, 1.00) | 0.86  (0.73, 1.00) |
| **FibrillarCPA-SHG** | 0.76  (0.66, 0.86） | 0.83  (0.74, 0.92) | 0.84  (0.75, 0.92) | 0.70  (0.59, 0.82) | 0.79  (0.70, 0.89) | 0.80  (0.71, 0.89) | 0.81  (0.65, 0.97) | 0.86  (0.72, 0.99) | 0.87  (0.74, 0.99) |
| **FibrillarAGG** | 0.78  (0.69, 0.88) | 0.82  (0.74, 0.91) | 0.85  (0.77, 0.92) | 0.72  (0.62, 0.83) | 0.82  (0.72, 0.92) | 0.81  (0.71, 0.90) | 0.80  (0.65, 0.95) | 0.89  (0.79, 1.00) | 0.89  (0.78, 1.00) |
| **NoXlinkF** | 0.77  (0.67, 0.87) | 0.82  (0.73, 0.91) | 0.83  (0.73, 0.92) | 0.71  (0.60, 0.83) | 0.82  (0.73, 0.90) | 0.82  (0.73, 0.91) | 0.78  (0.61, 0.96） | 0.84  (0.70, 0.97) | 0.85  (0.72, 0.98) |

*Portal CPA-SHG was set as the reference data. Abbreviation: SHG, total collagen proportionate area quantified by SHG/TPEF; Agg, aggregated collagen; NoStrS, number of collagen strings in septal area; NoThickStrS, number of thick strings in septal area; StrLengthS, the length of collagen strings in septal area; StrLengthSA, the length of aggregated collagen strings in septal area; StrWidthSA, the width of aggregated collagen strings in septal area; NoXlink, number of cross-linked collagen strings.
