# Supplementary figures and images for: SHG/TPEF-based image technology improves liver fibrosis assessment of minimally sized needle biopsies
Source: Hepatol Int. 2019 Jun 11;13(4):501–9. doi: 10.1007/s12072-019-09955-2 (PMC6661026; doi:10.1007/s12072-019-09955-2)

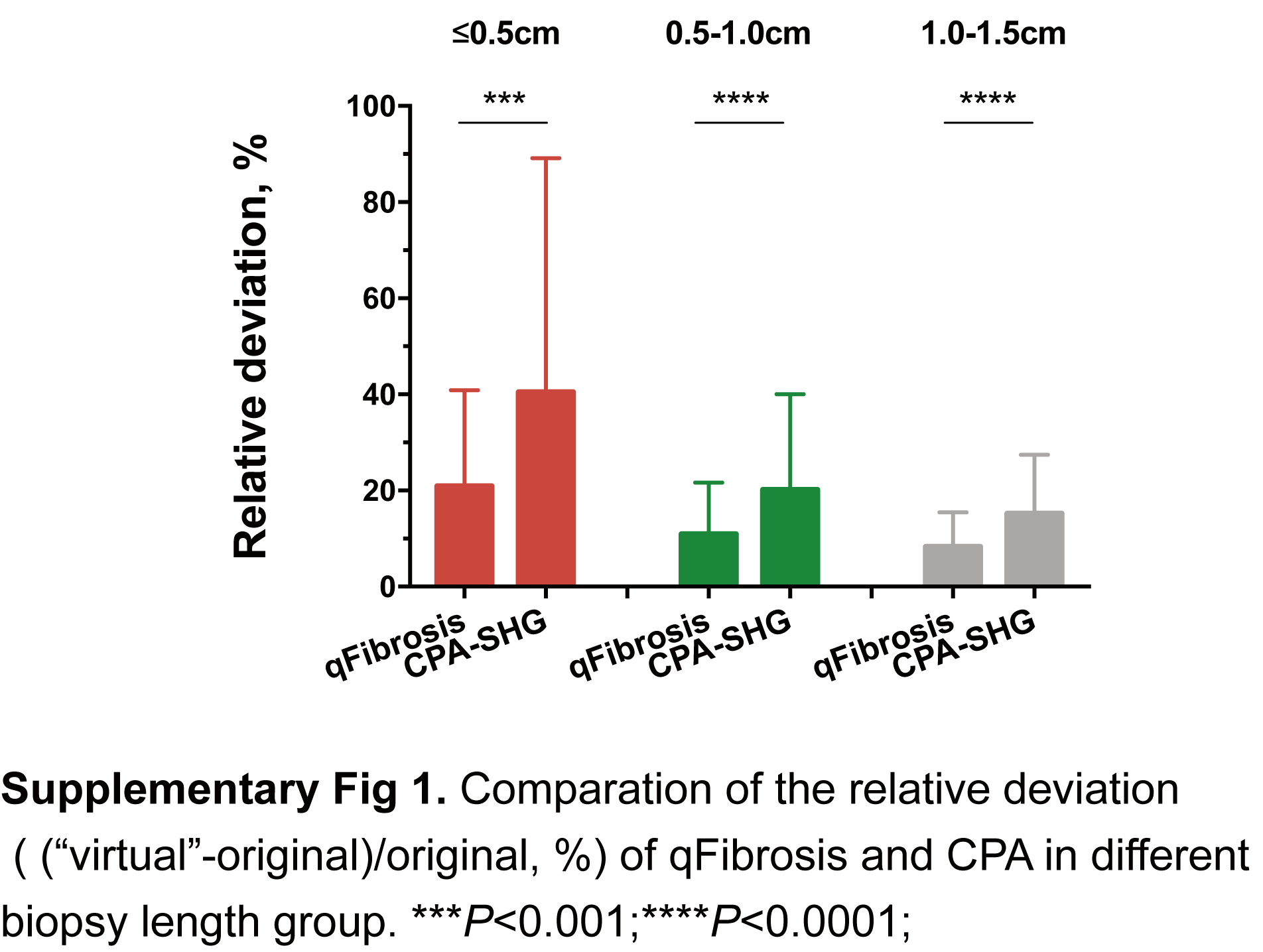

Supplement: Supplementary file 3 — Supplementary material 3 (TIFF 745 kb) [file 12072_2019_9955_MOESM3_ESM.tif]

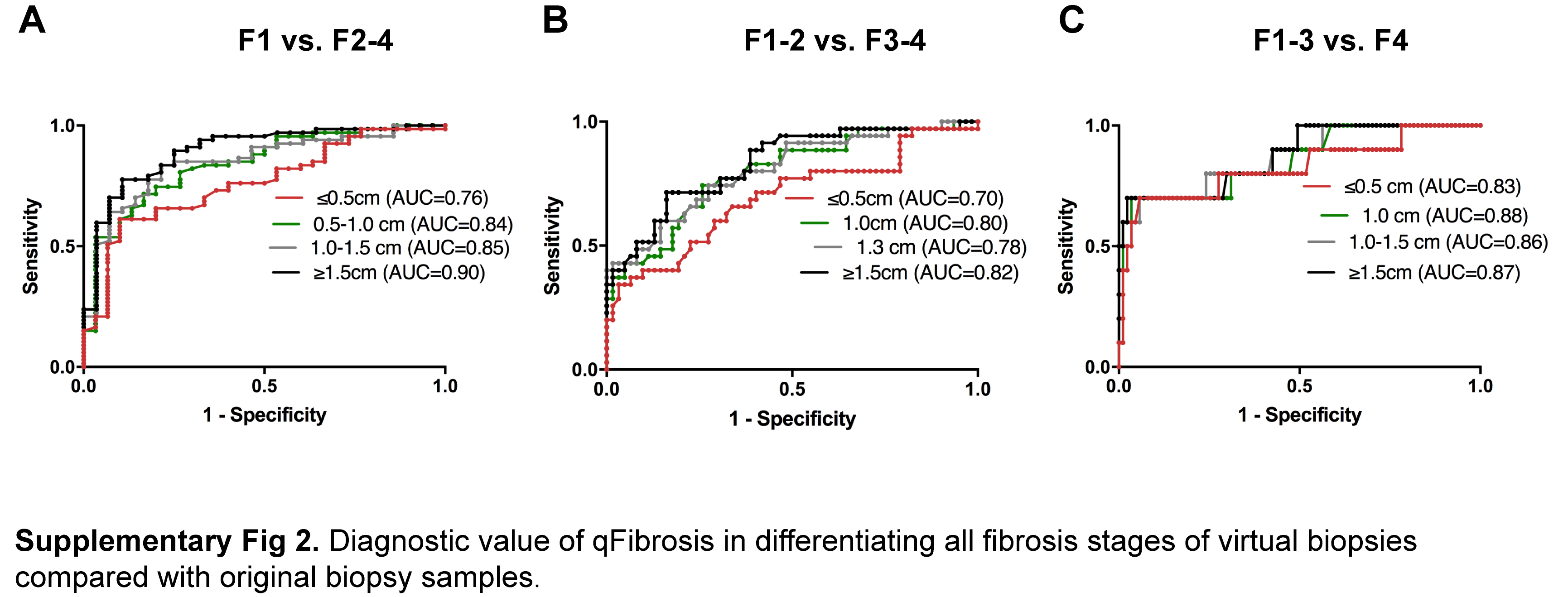

Supplement: Supplementary file 4 — Supplementary material 4 (TIFF 1734 kb) [file 12072_2019_9955_MOESM4_ESM.tif]

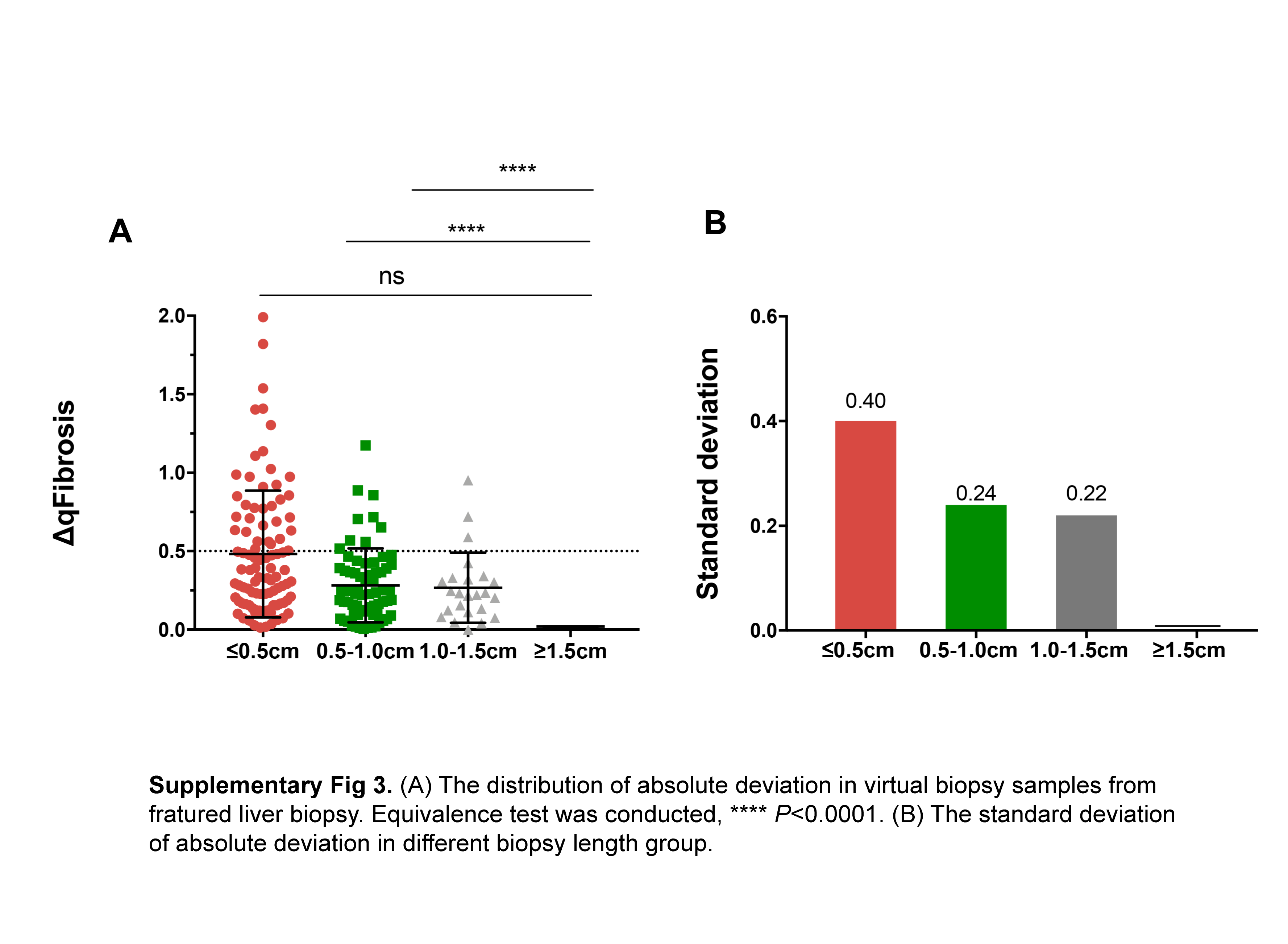

Supplement: Supplementary file 5 — Supplementary material 5 (TIFF 1635 kb) [file 12072_2019_9955_MOESM5_ESM.tif]

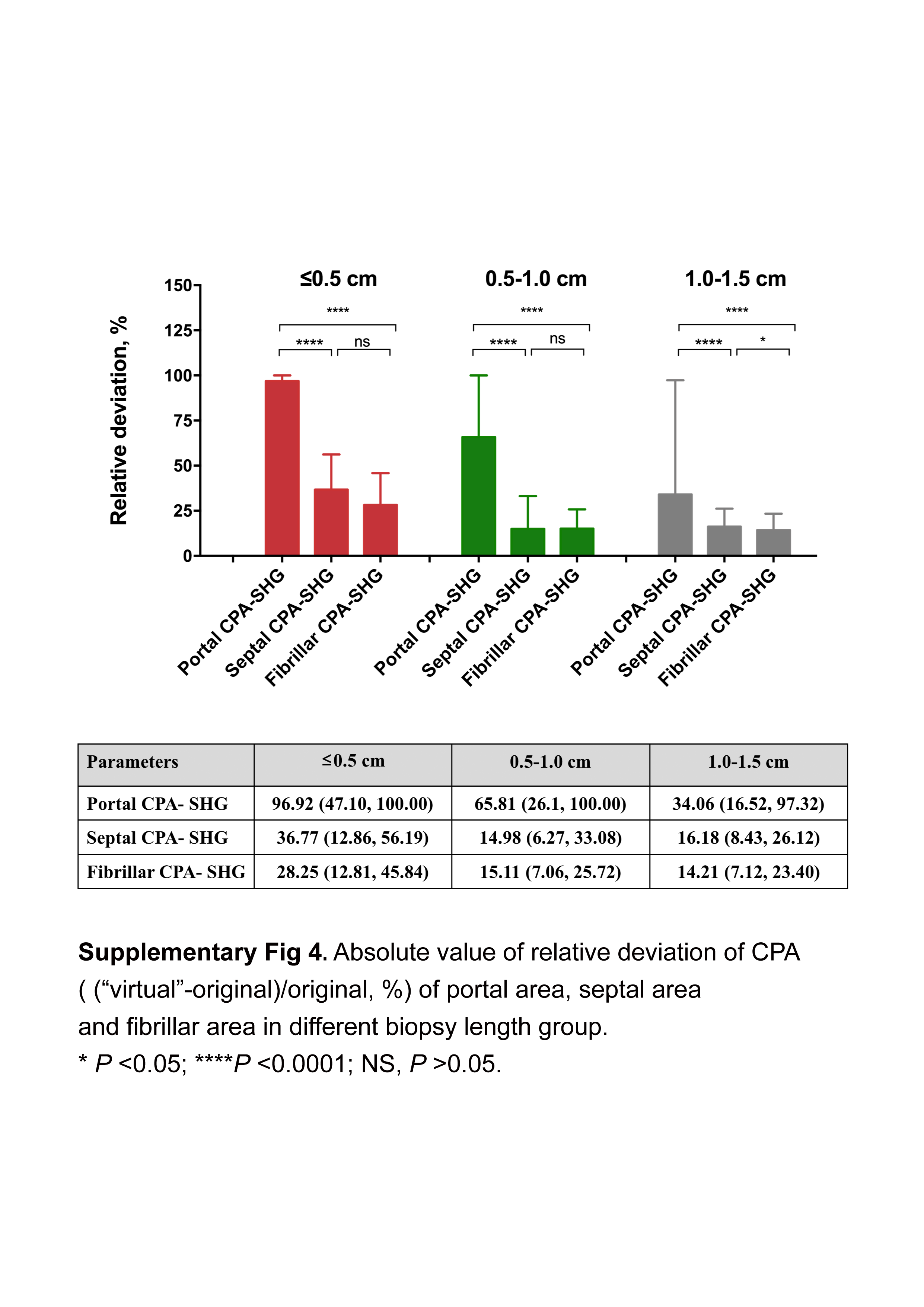

Supplement: Supplementary file 6 — Supplementary material 6 (TIFF 2289 kb) [file 12072_2019_9955_MOESM6_ESM.tif]

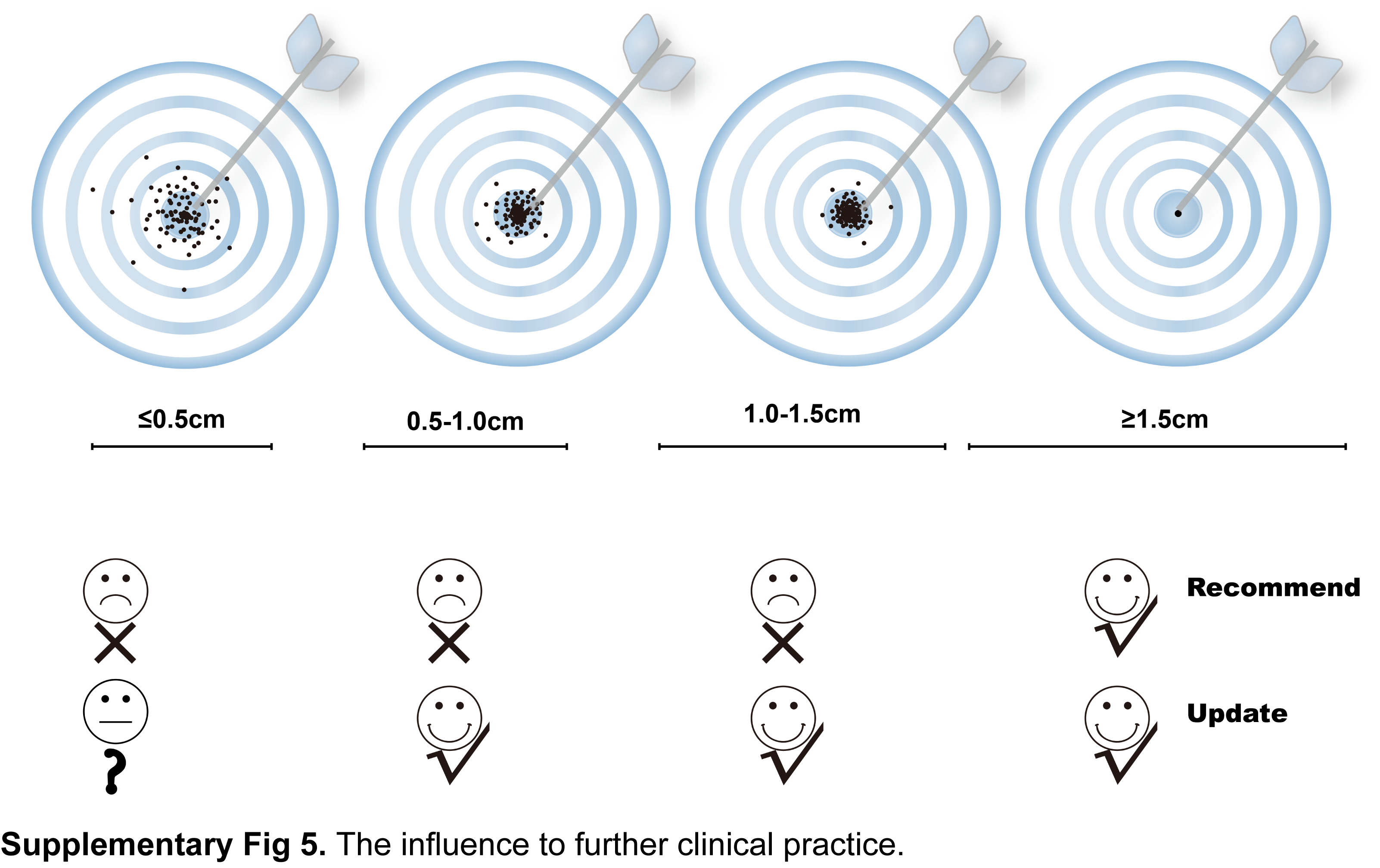

Supplement: Supplementary file 7 — Supplementary material 7 (TIFF 3384 kb) [file 12072_2019_9955_MOESM7_ESM.tif]
